# Influence of Temperature on the Mechanical Performance of Unidirectional Carbon Fiber Reinforced Polymer Straps

**DOI:** 10.3390/ma14081903

**Published:** 2021-04-11

**Authors:** Danijela Stankovic, Luke A. Bisby, Giovanni P. Terrasi

**Affiliations:** 1 School of Engineering, William Rankine Building, The University of Edinburgh, Thomas Bayes Road, King’s Buildings, Edinburgh EH9 3FG, UK; Luke.Bisby@ed.ac.uk (L.A.B.); Giovanni.Terrasi@empa.ch (G.P.T.); 2 Empa, Swiss Federal Laboratories for Materials Science and Technology Überland Str. 129, 8600 Dübendorf, Switzerland

**Keywords:** thermo-mechanical behavior, unidirectional (UD) composites, carbon fiber reinforced polymer (CFRP) straps, elevated temperature tensile properties, steady state thermal behavior, transient state thermal behavior

## Abstract

The performance of pretensioned, laminated, unidirectional (UD), carbon fiber reinforced polymer (CFRP) straps, that can potentially be used for example as bridge deck suspender cables or prestressed shear reinforcements for reinforced concrete slabs and beams, was investigated at elevated temperatures. This paper aims to elucidate the effects of elevated temperature specifically on the tensile performance of pretensioned, *pin-loaded* straps. Two types of tests are presented: (1) steady state thermal and (2) transient state thermal. Eight steady-state target temperatures in the range of 24 °C to 600 °C were chosen, based on results from dynamic mechanical thermal analysis (DMTA) and thermogravimetric analysis (TGA). Transient state thermal tests were performed at three sustained tensile load levels, namely 10, 15, and 20 kN, corresponding to 25%, 37%, and 50% of the ultimate tensile strength of the pin-loaded straps at ambient temperature. In general, the straps were able to retain about 50% of their ambient temperature ultimate tensile strength (UTS) at 365 °C.

## 1. Introduction

Carbon fiber reinforced polymers (CFRPs) have been extensively used in aerospace, automotive, and structural engineering applications for more than 50 years [1]. In engineering, the high strength-to-weight ratios and tensile rigidity facilitate their use as both reinforcing materials and in stand-alone applications [2,3,4]. Previous researchers have extensively documented the tensile behavior of unidirectional (UD) CFRP. For instance, the basic damage mechanisms of UD CFRP laminate plates under tensile loading have been documented by Talreja and Singh [5]; these include fiber breakages/splitting, fiber/matrix debonding, and microcracking in a plane transverse to the fiber direction. In terms of the tensile strength of unidirectional elements, it is generally assumed that fiber breakage is the dominant initiating failure mechanism. In contrast, when a UD CFRP element is not a composite plate, but a curved composite part, it has been shown that the governing failure mechanisms may differ from those mentioned above [6]. Under quasi-static tensile loading, the failure process may be a complex progression of ply cracking, delamination (initiated due to radial stresses caused by bending) and sudden fiber breakage [7,8]. 

In the context of civil/structural applications, CFRP elements have previously been used as suspension cables, and a detailed summary of applications is given by Wang et al. [9]. One simple (in terms of anchorage) fiber dominated tensile element is the pin-loaded, looped strap, which transfers tensile forces similarly to an individual chain link [8]. Such straps were first mechanically characterized by Conen in 1966 [10], who used UD glass fiber reinforced polyester plies to laminate looped straps. The first studies on pin-loaded laminated and non-laminated (thermoplastic matrix) CFRP straps were conducted by Winistörfer at Empa in late 1990s [11,12]. Winistörfer investigated matrices’ and fibers’ mechanical properties, different looping techniques, frictional properties, and stress concentration regions for the straps under tension and also reported a considerable increase in the efficiency of the load transfer with increasing pin radius (for non-laminated straps with a thermoplastic polymer matrix). Winistörfer’s results indicated that the non-laminated straps were creep resistant under sustained loads and were, thus, considered suitable for shear reinforcement of concrete structural elements. Lees et al. [13] subsequently experimentally demonstrated that using external prestressed non-laminated CFRP straps could be an effective means to enhance the shear capacity of reinforced concrete beams; results showed that concrete beams strengthened with non-laminated CFRP straps could achieve 38% increased load capacity as compared with unstrengthened beams. 

**Figure 1:**
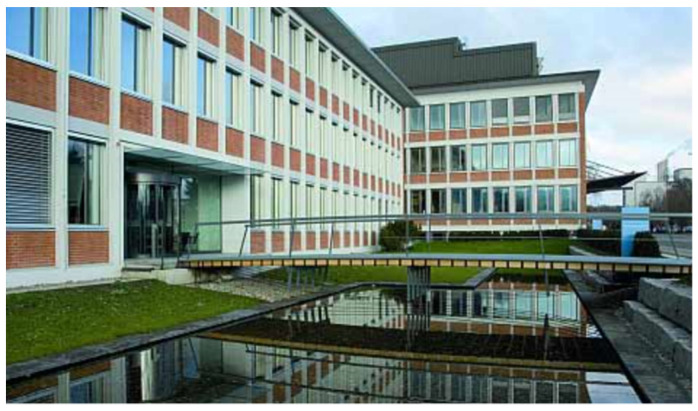
Empa bowstring arch footbridge [14]. Reprinted with permission from ref. [14]. Copyright 2009 Empa.

**Figure 2:**
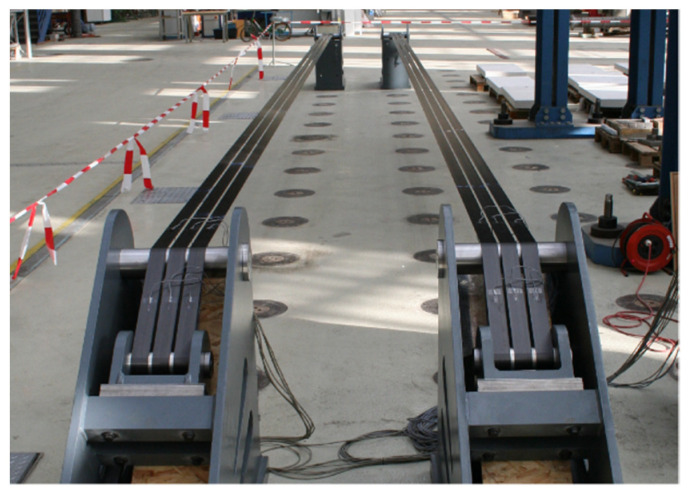
Stress-ribbon footbridge [15]. Reprinted with permission from ref. [15]. Copyright 2007 Ernst & Sohn Verlag für Architektur und technische Wissenschaften GmbH & Co. KG, Berlin.

**Figure 3:**
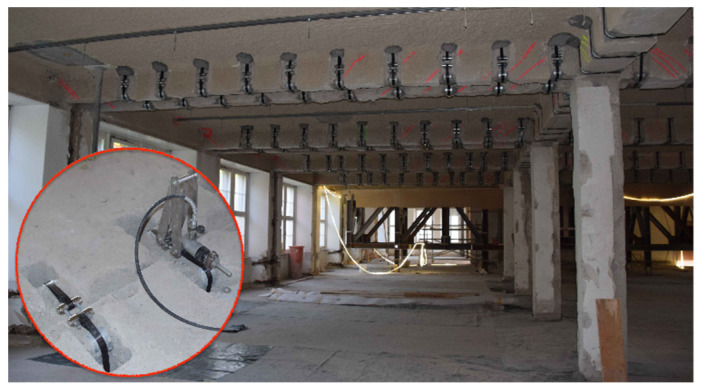
Slab reinforcement with prestressed non-laminated pin-loaded Straps (bottom left: prestress detail).

Practical applications and design concepts for pin-loaded non-laminated straps include the bowstring arch footbridge at The Swiss Federal Laboratories for Material Science and Technology (Empa) [14], which represents the application of such a configuration in timber bridge construction (Figure 1). A stress-ribbon bridge has also been realized by Schlaich and Bleicher [15] (Figure 2), wherein non-laminated CFRP straps acted as ribbons; in total six ribbons, prestressed by receiving a 6.5% elongation, were used and were able to carry a tensile force of 530 kN. A further application of non-laminated prestressed CFRP straps used them as prestressed shear reinforcements for concrete beams (Figure 3) [4] in the former Bank Leu premises in Zurich (2019). In total, 1080 non-laminated CFRP straps, pretensioned to 100 kN each, were used.

**Figure 4:**
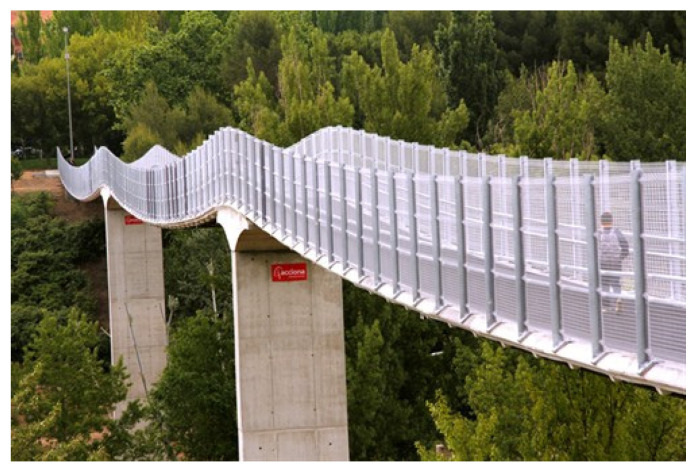
Stress ribbon footbridge in Cuenca, Spain (photo by ACCIONA [19]).

Most CFRP strap applications in mechanical engineering [10] and civil structures [16], however, focus on laminated straps, i.e., straps which are layer-wise bonded by hardening their thermoset matrix. The reasons for this are the lower material cost of the epoxy-matrix based UD prepreg (compared to thermoplastic prepregs used in non-laminated straps), the simpler production technique by tape winding and the better protection of the laminated cross-section against transverse loads and impact. A three-span footbridge built in 2011 in Cuenca (Figure 4), Spain, by ACCIONA consists of cables made from laminated CFRP straps which are loaded on steel pins [17]. The choice of the laminated CFRP straps was mainly due to them having greater stiffness and smaller creep when compared with non-laminated CFRP straps. Further examples of laminated and non-laminated CFRP straps, as well as other CFRP cable configurations, are given by Liu et al. [18]. The main disadvantage of the laminated straps is their lower tensile efficiency (due to stress concentrations, lack of frictional effects between plies, etc.) than the more expensive non-laminated straps (in the range of 20–30% lower efficiency, depending on the straps’ geometries [11]). The stress distributions in pin-loaded laminated straps under static tension was studied in [8], and the critical failure-triggering region was identified as the transition region between the pin and the straight part (i.e., the ‘shaft’). 

Baschnagel et al. further investigated the fretting fatigue behavior of laminated CFRP straps intended for use as bridge suspenders [20,21]. In their work, the residual mechanical properties of CFRP straps subjected to tensile fatigue loading with a frequency of 10 Hz (R = 0.1) were assessed and the fatigue limit stress was observed to be reached after 3 million loading cycles at 46% of their ultimate tensile strength (UTS) (i.e., 750 MPa). The critical failure region at the vertex area of the pin/strap contact interface was deemed to be influenced by stress concentrations. The reported damage mode was consistently delamination that initiated at the ends of the overlapping zone and progressed towards the vertex area; final failure was explosive and sudden in nature. Overall, Baschnagel et al.’s results indicated that the looped CFRP tension members can compete well with the equivalent steel members, since they exhibited superior fatigue strength, superior specific strength and stiffness, and high corrosion resistance.

However, because environmental changes, particularly changes in temperature, can affect the endurance and load bearing capacity of structural components, it is crucial to consider the influence of higher temperatures on the mechanical performance of the component. When considering CFRP pin-loaded straps as bridge hanger cables, for instance, fire incidents like that on the New Little Belt Bridge in 2013 [22], which primarily involved steel and concrete structural cables/members, need to be considered by designers [23,24]. Composite materials, such as CFRP, are increasingly being used in civil engineering structures which may be at risk of fire [25]. High temperature conditions can drastically affect the mechanical performance of CFRPs, as pointed out by Mahieux [26], who described thermal degradation of FRPs through four regions of the master curve (modulus-temperature). The softening behavior of composite elements at elevated temperatures is mainly dominated by the properties of the polymer matrix, and, in order to design a sufficiently safe load-bearing structure using FRP, mechanical property degradation over a broad temperature range must be known [27]. 

Comparatively little information is currently available on the degradation of mechanical properties and damage mechanisms of heat-exposed UD CFRP specimens in the form of either coupons, rods, strips, or tendons under tension [28,29,30,31]. Feih and Mouritz [28] investigated the tensile strength and fiber modulus of pure PAN-based T700 fibers and found that the fibers retained 50% of their room temperature UTS when exposed to temperatures between 400 and 600 °C in air. They also showed that when embedded with epoxy resin, the fibers decomposed (by oxidation) on the heated surface and became thinner in diameter at temperatures above 500 °C in air. The carbon fibers in depth within the CFRP remained intact because air could not diffuse into the composite due to the out-gassing of volatiles generated by decomposition of the polymer matrix. 

Terrasi et al. [30] investigated the tensile strength of UD high-modulus pitch based CFRP rods at elevated temperatures and reported an average tensile strength of 644 MPa at 570 °C (this being 46% of room temperature UTS). For UD CFRP tendons (T700 fibers, epoxy matrix, 65% fiber volume content), Zhou et al. [31] found, from steady state tests, a 50% reduction of the ambient temperature UTS at 324 °C. In addition, from transient thermal tests they reported that the time to failure of CFRP tendons increased with the decrease of loading level, with the temperature corresponding to a 50% reduction of ambient temperature UTS being 341 °C. The main observation in all the studies was that the specimens could continue to carry substantial applied loads even after polymer matrix softening and decomposition; eventually they failed due to fiber breakage. 

The work presented in the current paper aims to provide insights into the mechanical performance of scaled-down models of a pin/CFRP strap system under tensile loading at elevated temperatures. The loading and temperature conditions are representative of those likely to be experienced during an accidental fire. The motivation behind this effort is the lack of knowledge on the high temperature tensile behavior of pin-loaded laminated CFRP straps, knowledge which is of paramount importance in order to be able to confidently design fire resilient CFRP straps used for applications, like bridge hanger cables or shear strengthening of concrete beams and slabs [13]. The static behavior of pretensioned, laminated, pin-loaded CFRP straps was tested under both ambient and elevated temperatures. The elevated temperature experiments were both steady state thermal and transient state thermal. In the former, eight target temperatures in the range of 24 °C to 600 °C were selected, based on results from dynamic mechanical thermal analysis (DMTA), differential scanning calorimetry (DSC), and thermogravimetric analysis (TGA). The transient state thermal case consisted of three different tensile load levels: 10 kN, 15 kN, and 20 kN, corresponding to 25%, 37%, and 50% of their ambient temperature ultimate tensile strength, respectively. Once the target load was reached, the temperature was increased from 24 °C to 600 °C until ultimate failure of the strap.

## 2. Material Characterization and Manufacture

### 2.1. Materials 

Two main components were used in the present study, namely titanium pins and CFRP straps. The titanium pins (Ti-6Al-4V, Grade 5, Narrowboat Way, Hurst Business Park, Brierley Hill, West Midlands, UK. [32]), as well as the material of the straps, in the form of a continuous unidirectional carbon prepreg tape (fibers: IMS60 E13 24K 830tex (Wuppertal, Germany) [33]; epoxy resin: XB 3515/Aradur^®^ 5021 by Huntsman (Basel, Switzerland) [34]), were supplied by CarboLink Ltd (Fehraltorf, Switzerland). Properties of both components are given in Table 1.

### 2.2. Characterization 

Characterization of the composite material of the straps is presented in this section. To observe the mass loss under heating in oxidative (air) and inert (nitrogen) atmospheres thermogravimetric analysis (TGA) was performed for the manufactured straps. For brevity, only the results for samples heated in air will be shown, since inside the environmental chamber used for the elevated temperature tensile tests the components were exposed to air. A Mettler Toledo thermogravimetric analyzer with a high temperature furnace was used for CFRP strap samples and a TG 209F1 Libra^®^ thermogravimetric analyzer was used for neat epoxy resin samples. Reusable aluminium oxide crucibles without lids were used for the TGA measurements. 

**Figure 5:**
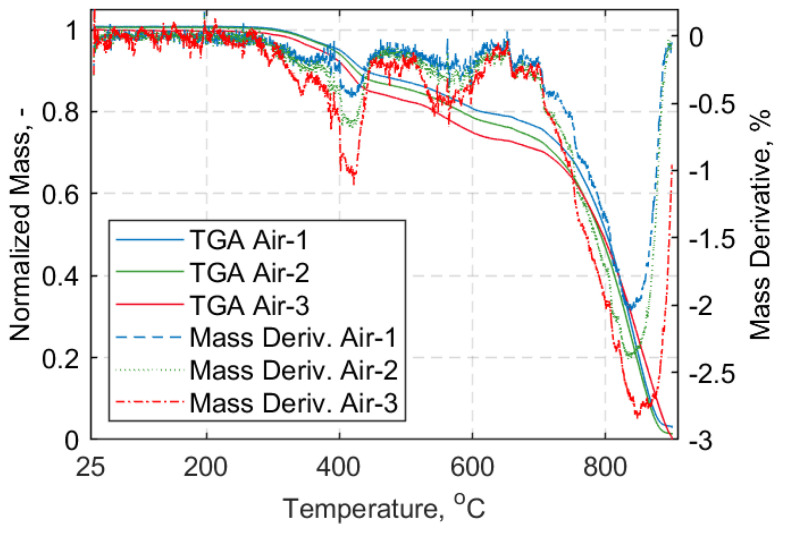
(**Left**): Normalized mass loss curve vs. temperature (°C). (**Right**): Mass derivative curve (%) vs. temperature (°C); air atmosphere for carbon fiber reinforced polymer (CFRP) straps.

**Figure 6:**
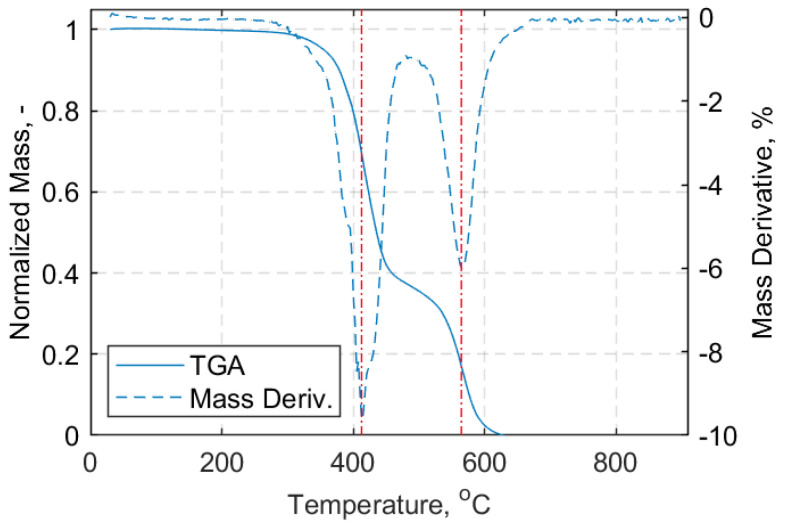
(**Left**): Normalized mass loss curve vs. temperature (°C). (**Right**): Mass derivative curve (%) vs. temperature (°C); air atmosphere for neat epoxy resin.

For all samples, a temperature range between 30 °C and 900 °C, with a heating rate of 10 °C/min, was used. The resultant mass loss curves are given in Figure 5 and Figure 6 for the composite material of the straps and the neat epoxy resin, respectively. Subsequently, the fiber volume fraction (FVF) of the cured composite strap material was determined using a standard ‘burn-off’ procedure. Five samples were cut from the straight shaft of the straps (approximately 1 mm × 20 mm) and tested according to ASTM D3171-15 standard test method [35]. The samples were all weighed using a 0.1 mg precision scale in a stable lab temperature of 22 °C using an Ohaus Adventurer AX324 analytical balance. The density of the composite samples (ρ) was obtained, as described in the Ohaus^TM^ Density Determination kit manual (Shanghai, China). The ‘burn-off’ tests were performed using a Nabertherm-L 15/11 muffle furnace at 480 °C for 2 h, and Equations (1)–(5) of the ASTM D3171-15 standard [35] following Test Method I, Procedure G, were used. The results of the weighed Haldenwanger porcelain crucibles (Fisher Scientific, Bishop Meadow Road, Loughborough, Leicestershire, LE11 5RG, UK) and the composite samples prior to and after the burn-off test are presented in Table A1 of Appendix A. The calculated values for FVF (*V_f_*), matrix volume fraction (*V_m_*) and void volume fraction (*V_v_*), as well as the calculated density of each sample, are given in Table 2, alongside the average values and their standard deviations. An average fiber volume fraction of 59.3% was obtained, which is similar to the value of 60% for the carbon fiber reinforced epoxy prepreg material issued by the prepreg’s producer, Carbo-Link Ltd (Fehraltorf, Switzerland). It is noteworthy that the burn-off procedure usually carries an error in the range of 2–3% compared with, e.g., a chemical digestion procedure [35]. 

**Figure 7:**
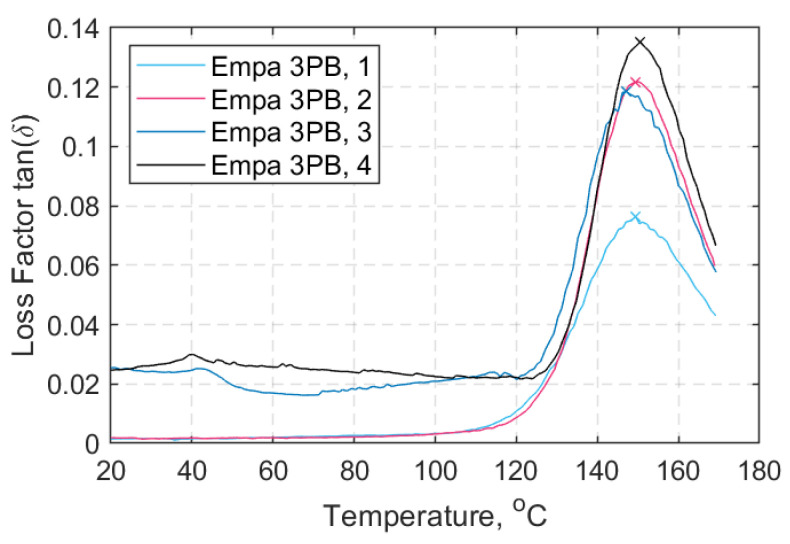
Loss factor tanδ over temperature (°C)—all dynamic mechanical thermal analysis (DMTA) samples.

Dynamic mechanical thermal analysis (DMTA) and differential scanning calorimetry (DSC) were additionally performed for the composite material of the straps to further characterize their physical properties. The glass transition temperature (*T_g_*) was obtained using both DMTA and DSC. Four DMTA samples were tested under a three-point-bending (3PB) mode and frequency *f* = 10 Hz using a thermal analyzer EPLEXOR 500 (Gabo Qualimeter GmbH, Ahlden/Aller, Germany); the samples were cut from the straight shaft length of the pretensioned straps used in this study. The temperature range was between −30 and 170 °C, with a heating rate of 2 °C/min. ‘Method B’ according to ISO standard 6721 [36] was followed to obtain the *T_g_* values (i.e., by taking the peak value of the loss factor, tanδ). Samples 1 and 2 prior to testing had a sagging-like shape, while samples 3 and 4 had a hogging-like shape. The reason for the samples not being straight prior to testing is attributed to the presence of residual stresses due to pretensioning of the straps during their manufacturing process (these are described elsewhere). Figure 7 shows the resultant DMTA curves with the tanδ peaks indicated in each case. It is noteworthy that the *T_g_* values according to a tanδ peak-definition are usually higher than those derived from a peak in the loss modulus curve [36]. It is also evident that the *T_g_* onset takes place at a lower temperature between 130 and 140 °C, as defined by the intercept of the tangents below *T_g_* and the slopes of the loss factor curves. A similar behavior of the glass transition of a two-part epoxy adhesive for structural strengthening has previously been reported by Stratford and Bisby [37], who defined *T_g_* as the tanδ-peak but noted that the stiffness reduction can take place at temperatures of up to 20 °C or more below this *T_g_*. 

**Figure 8:**
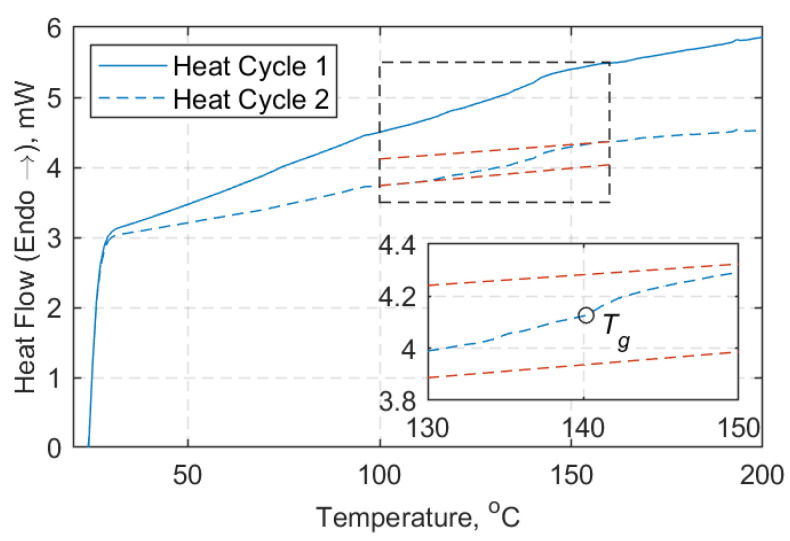
Heat flow (mW) over temperature (°C): Representative differential scanning calorimetry (DSC) curve.

**Figure A1:**
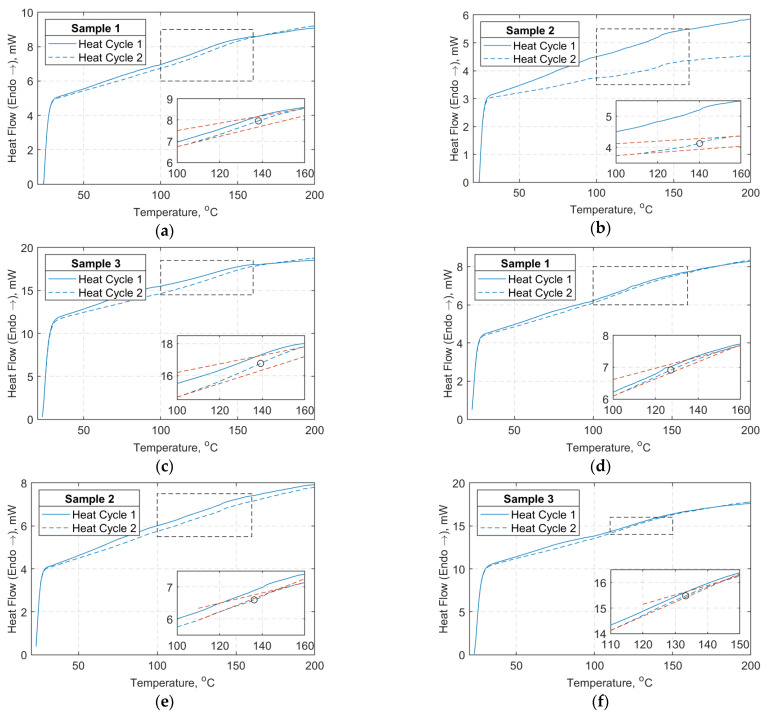
DSC plots (heat flow, mW vs. temperature, °C) for composite material of the straps: (**a**–**c**) are the samples extracted from the middle shaft length of the straps, while (**d**–**f**) are the samples from the curvature region of the straps.

Six DSC results were obtained using a DSC 8000 double-furnace by PerkinElmer^©^ (Waltham, MA 002451, USA), for which three samples were extracted from the middle of the straight shaft length of the straps and three from the curved region of the straps. Aluminium pans with lids and a minimum weight of 10 mg per sample were used. A heat-cool-heat cycle was followed, for which each sample was heated from 20 °C to 200 °C, cooled down to 20 °C, and then increased up to 200 °C at a heating rate of 20 °C/min, as described in ASTM D3418-15 [38]. A representative DSC curve is illustrated in Figure 8, where the midpoint temperature (*T_g_*) is also shown. The difference observed between the average *T_g_* values obtained with DMTA and DSC can be attributed to the nature of the tests (see Table 3). Further results are given in Appendix A, Figure A1. 

### 2.3. Manufacture 

**Figure 9:**
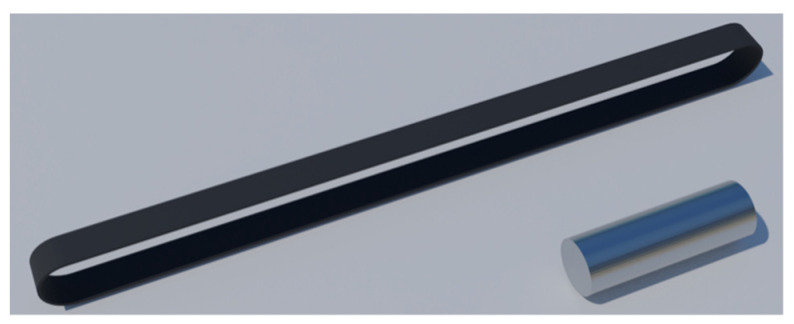
Laminated strap and titanium pin.

**Figure 10:**
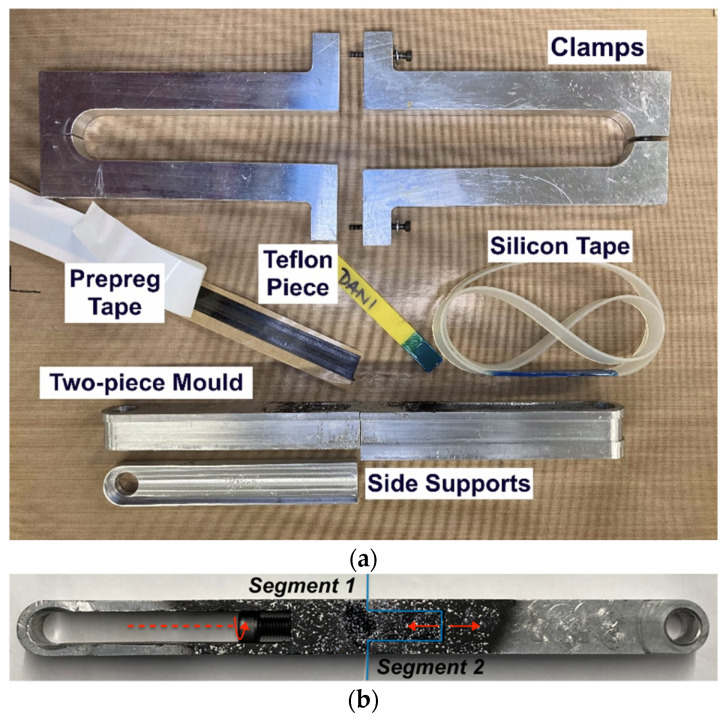
Pretensioning mold: (**a**) Parts used in manufacturing; (**b**) side view of two-piece mold.

**Figure 11:**
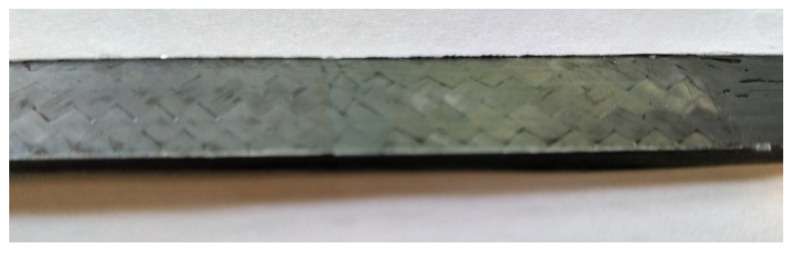
±45° carbon twill ply.

The bespoke titanium pins were supplied in specific dimensions (Table 4) and known material characteristics and are shown in Figure 9. The straps were manufactured by winding the continuous UD carbon prepreg tape around a two-piece aluminium mold that was then enclosed by aluminium clamps. Between the prepreg tape and the clamps, a silicon tape was placed to aid in the demolding process. The mold was designed in such a way as to be able to pretension the straps prior to and during curing. Two segments comprise the mold, as illustrated in Figure 10. Initially, the first segment was joined with the second segment and two removable side supports were placed on one side of the assembled mold. Following that, the carbon (prepreg) tape was wound around the two-piece mold; an additional ±45° carbon twill ply was placed at the extremities of the carbon tape (see Figure 11). The dimensions for both the pin and the strap used in the current study are given in Table 4.

The next step was to apply an initial axial tensile strain of approximately 0.20% by screwing an M8 bolt within the mold; this drives the two mold segments apart through a pushing cylinder. This corresponds to a preload of about 10% of the average tensile failure load of the straps. The preload level was considered to be active throughout the resin curing—identical measurements of the physical gap generated between the two mold segments were taken prior to and after the curing of the straps and no change was detected. The straps were cured following the guidance given in the prepreg manufacturer’s datasheet [34]. The straps first remained at 120 °C for 1 h followed by 2 h at 140 °C, after which the curing cycle ended. The preload approximation is explained in detail in Section 3. Finally, the clamps fully enclosed the mold. A thin silicon cover was placed between the carbon tape and the outer clamps surface. The main intent of using the pretensioning mold—besides resulting in residual stresses in the cured composite—was that it would allow a better fiber alignment in the finished composite. In addition, the side of the pretensioning mold was spray-painted black and speckled with a random white pattern so that the displacement field could be monitored using image correlation (see Figure 10b).

## 3. Preload Estimation & Experimental Set-Up

### 3.1. Preload Experimental Estimation 

**Figure 12:**
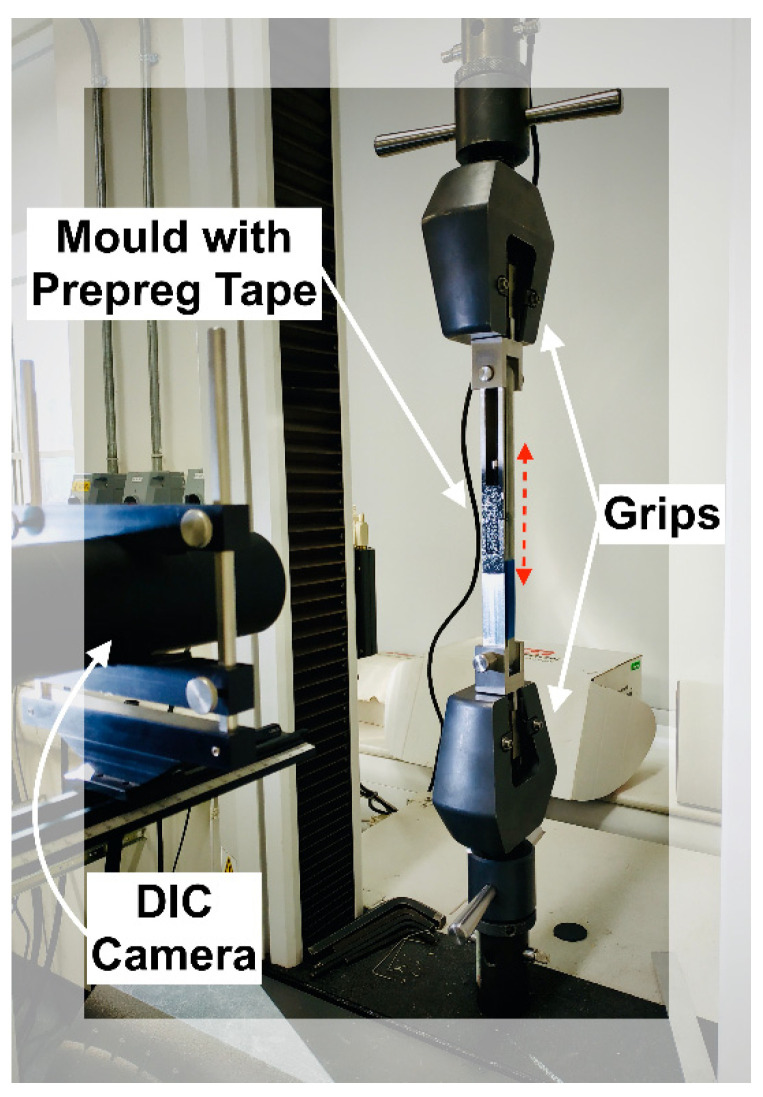
Experimental set-up for preload estimation.

**Figure 13:**
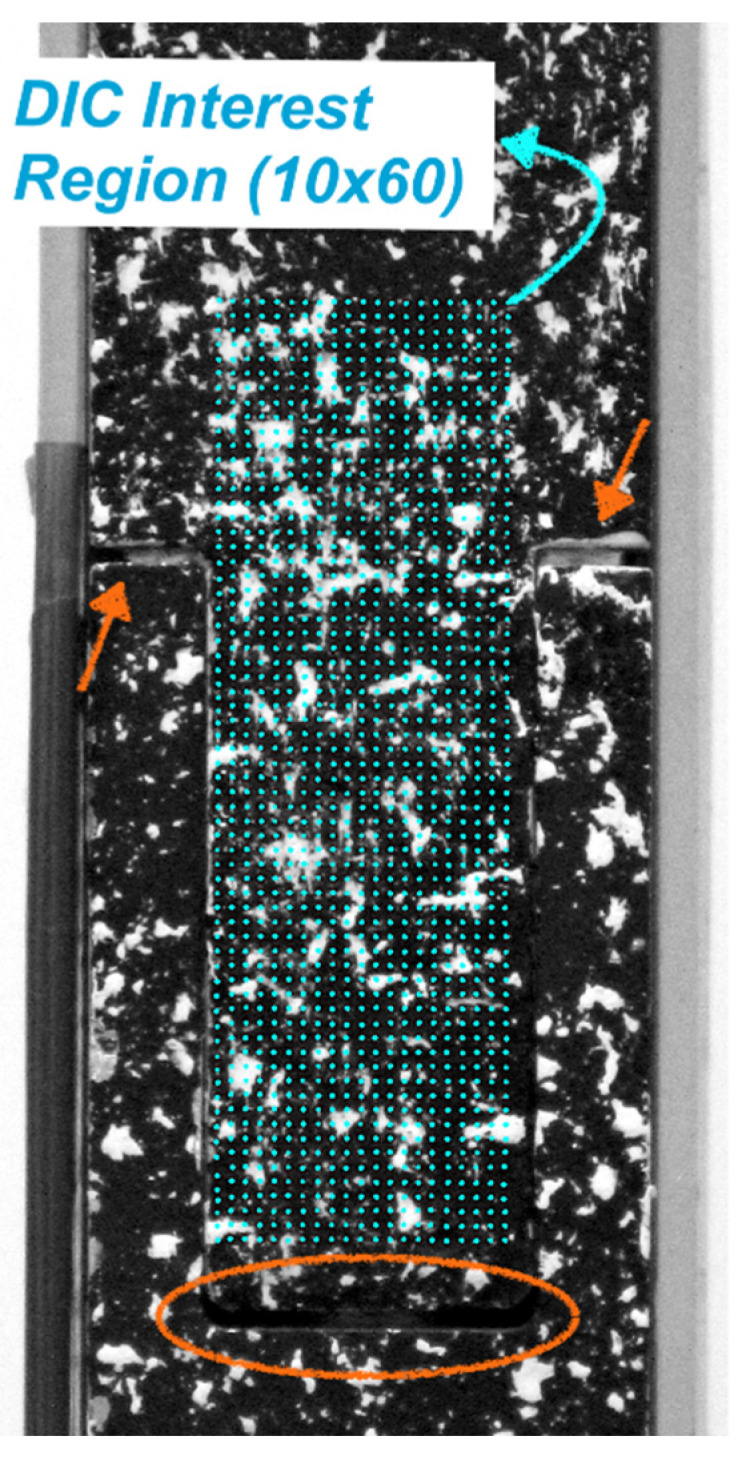
Detailed region of interest for digital image correlation (DIC) measurements and gap generation due to preload.

An approximation for the preload (pretension) applied to the uncured straps was measured experimentally, and the set-up is shown in Figure 12. The mold, which was first wrapped with six plies of UD carbon prepreg tape, was placed on an Instron 3369 (Norwood, MA, USA) testing frame with a 10 kN load cell using custom fixtures, and loaded until reaching 10% of the average ambient temperature failure load (*F_max_*) of the cured straps. A constant crosshead displacement rate of 0.5 mm/min was selected for the test. Throughout the loading cycle, displacement data were acquired using a Canon EOS 600D (Hong Kong, China) camera (indicated as “DIC Camera” in Figure 12) and a remote trigger timer with a consistent sampling rate of 0.2 Hz. Three straps were tested this way and the data were analyzed using Python 3 (pydic, Python Software Foundation [39]) and MATLAB R2019b. The region of interest used in the digital image correlation (DIC) analysis for the computation of displacements is shown in Figure 13 and was maintained consistent for all three tests. Once 0.1 *F_max_* was reached, the test was stopped. The M8 bolt was tightened to maintain the preload and the gap that was generated between the two segments of the mold was measured with a Vernier caliper at four different locations. The top left and top right gaps are indicated with arrows and the middle gap is the circled region in Figure 13. Subsequently the mold was removed from the Instron testing frame, enclosed by the clamps and placed in the oven for curing (as described in Section 2.3). It should be noted that the gap(s) generated between the two segments of the mold indicated that the preload remained active (see Figure 13).

**Figure 14:**
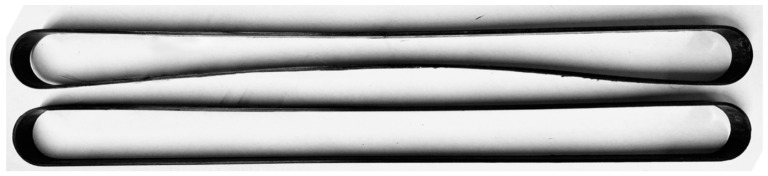
Shape change of prestressed straps (**top**) vs. non-prestressed straps (**bottom**).

**Figure 15:**
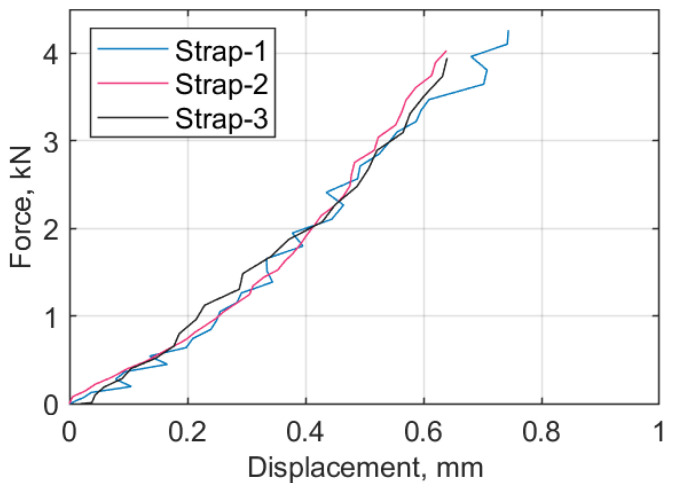
Load vs. (DIC) displacement curves for straps loaded up to 0.1 *F_max_*.

It has been observed that pretensioning the straps resulted in a minor change of geometry in the cured and de-molded samples, as illustrated in Figure 14, where an inwards bowing of the shafts of the straps is evident for those that were prestressed during curing. This behavior was not evident when the straps were made with a non-pretensioning mold. The results obtained from the three uncured straps are illustrated in Figure 15, where the axial load versus displacement plots obtained using DIC are presented. The final displacement values obtained with DIC, δ_max_ (gap between the two mold segments), and in-situ measurements with the caliper are presented in Table 5. 

The analysis above indicates that 10% of the *F_max_* was equivalent to 0.91 mm (average caliper measurement) spacing between the two mold segments; this was kept constant for all straps in the manufacturing process in order to apply the same preload. In all cases, physical measurements were taken with a caliper rather than utilizing DIC, for simplicity and to increase the rate of specimen manufacture.

### 3.2. Test Set-Up 

**Figure 16:**
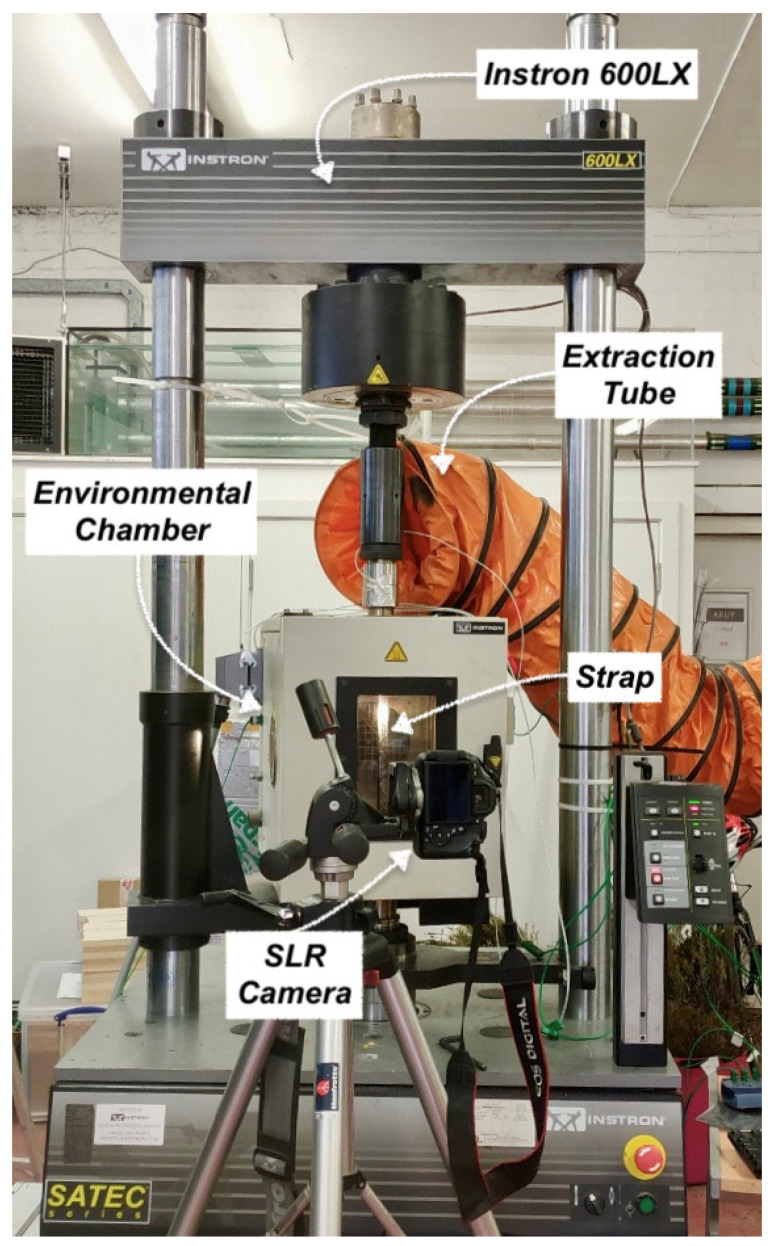
Ambient and elevated temperatures test set-up.

**Figure 17:**
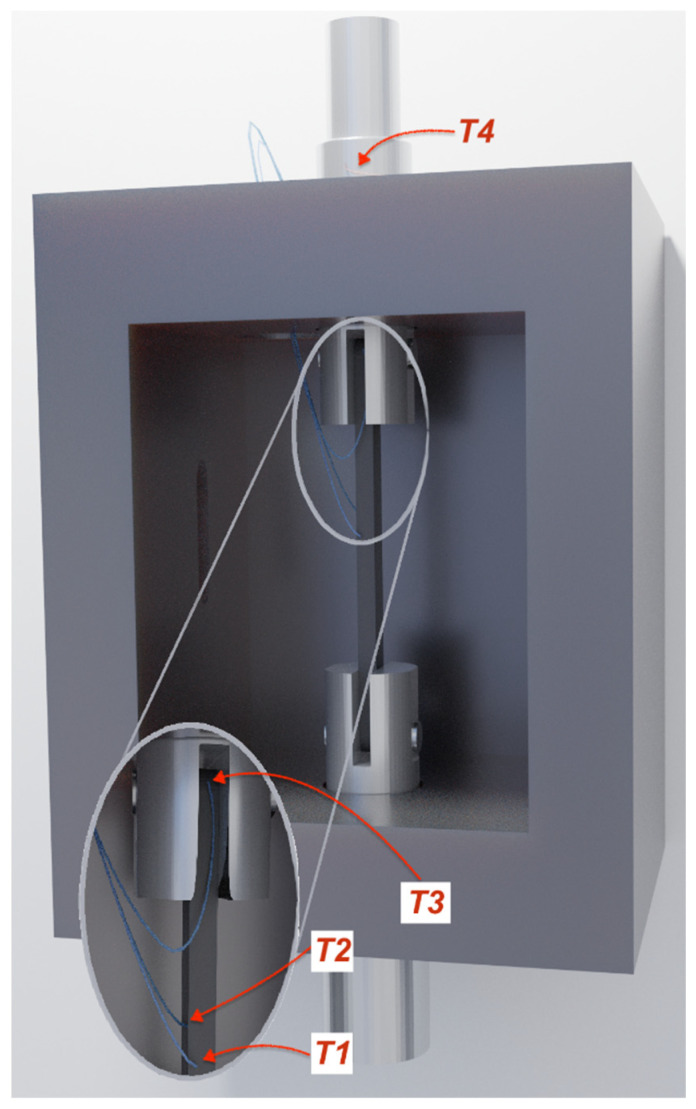
Position of thermocouples in chamber.

The main test set-up is illustrated in Figure 16 and was used for both ambient and elevated temperature experiments. An Instron 600LX hydraulic universal testing machine with an integrated Instron CP103790 environmental chamber was used (High Wycombe, UK). The load capacity of the machine is 600 kN, and the upper temperature limit of the environmental chamber is 600 °C. The strain and deformation fields were monitored using DIC analysis with a Canon EOS 600D camera and a remote trigger timer at a sampling rate of 0.3 Hz. The straps were speckled using high temperature paint to assist with the strain mapping. The image analysis was used to approximate the elastic modulus of the straps at different temperatures. The straps were pin-loaded on custom made stainless steel grips that were screw-fixed to the Instron; the CFRP strap, the titanium pins, and part of the grips were placed inside the environmental chamber, as illustrated in Figure 16 and Figure 17. Four Type-K thermocouples were used in both the steady state thermal (SS) and transient state thermal (TS) tests, the locations of which are shown in Figure 17. Thermocouples 1 (T1) and 2 (T2) were placed inside the chamber at the center of the front straight shaft of the strap and the center of the rear straight shaft of the strap (closer to the heating elements and fan heater), respectively. Thermocouple 3 (T3) was initially placed on the top edge of the top anchorage/loading pin, but, due to disbonding after about 170 °C, it was decided to place it on the vertex of the strap; Thermocouple 4 (T4) was placed outside of the chamber on the top pull rod.

**Figure 18:**
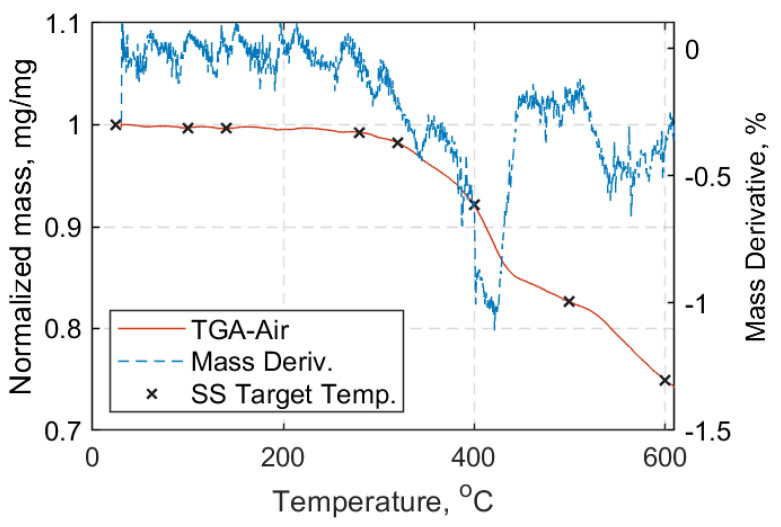
Representative thermogravimetric analysis (TGA) curve in air atmosphere for CFRP Straps with steady state (SS) target temperatures denoted.

In the SS experiments, the straps were initially loaded using a load hold mode at 0.5 kN tensile load until the target temperature was reached. The straps remained at the target temperature for a further 10 min to ensure an even distribution of temperature in the pin-strap system. After 10 min, the hold mode was switched to displacement control mode and the straps were loaded at a displacement rate of 2 mm/min until failure; five tests were performed for each temperature. The eight target temperatures selected were: 24 °C (ambient), 100 °C, 140 °C, 280 °C, 320 °C, 400 °C, 500 °C, and 600 °C. The choice of target temperatures was based upon the TGA samples tested in air, and aimed for those temperatures where mass loss was more pronounced and/or transition regions. A representative curve for an air exposed TGA sample, with the target testing temperatures also denoted, is given in Figure 18. For the TS cases, the straps were loaded with displacement control and a rate of 2 mm/min until the target load (10 kN, 15 kN, or 20 kN) was reached. The specified load level was maintained constant under load control while the temperature was increased up to 600 °C, or until failure. For each TS case, three tests were performed. In both SS and TS tests, the heating rate was 10 °C/min.

## 4. Results

### 4.1. Material Characterization 

The degradation mechanisms and the interpretation of the material reactions are complex. In Figure 5, three distinctive stages (peaks) are evident; first, above 340 °C and with a clear peak at about 410 °C, the decomposition of the epoxy resin takes place. This continues up to about 600 °C. Above 700 °C the oxidation of the fibers begins, and peaks at about 840 °C. An average FVF of 59.33 ± 2.95% was found from the five burn-off tests—marginally lower than the one the supplier [4] guarantees for its prepregs (60–65%). Similarly, the average matrix volume fraction (*V_m_*) and the void volume content (*V_v_*) were found to be 34.90 ± 1.37% and 5.78 ± 2.55%, respectively. Although, *V_v_* is high it is associated with the uncertainty of the burn-off procedure, as mentioned in ASTM D3171-15 [35]. The average *T_g_* was found to be 149.2 ± 1.42 °C and 135.7 ± 4.85 °C via DMTA and DSC tests, respectively, as shown in Table 3. The difference between the average *T_g_* values can be attributed to the nature of the tests and the state of the samples prior to testing. It is notable that the DSC samples for which the extracted location was the middle shaft length, experienced slightly overall higher *T_g_* values. This can possibly be attributed to the manufacturing process as the middle shaft region experiences lower pressures than the curvature region enclosed by the clamps. Overall, the *T_g_* is in the expected range of 140 °C, as indicated in the data sheet of the hot melt epoxy for the curing cycle applied [34], and the decomposition temperature of the epoxy matrix begins at about 410 °C.

### 4.2. Steady State Thermal (SS) Results

As already mentioned, for the SS conditions five tests per target temperature were performed. In this section, for reasons of brevity, only one test per category is presented with further test details provided in Appendix B. The specimen naming notation denotes:

- SS-Steady State;
- S-Standard Strap Models;
- P-Prestressed; and
- 24 °C, 100 °C, 140 °C, …, 600 °C for each temperature case.

**Figure 19:**
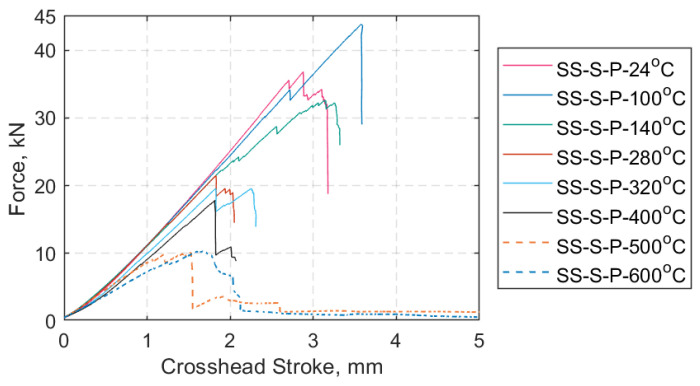
Force (kN) vs. crosshead stroke (mm) representative curves for each SS case.

**Figure 20:**
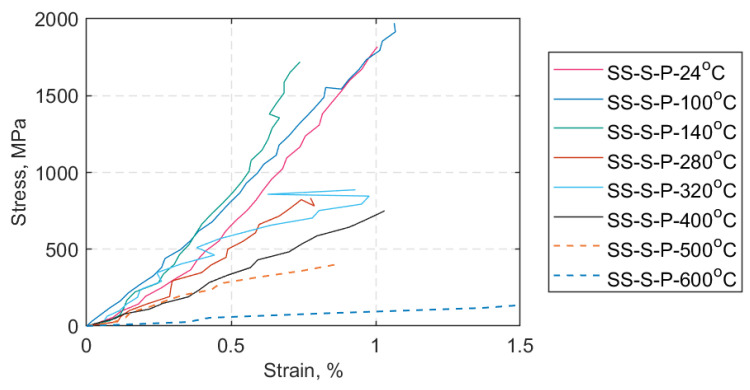
Stress (MPa) vs. strain (%) representative curves for each SS case.

The axial force versus crosshead displacement representative curves for each SS target temperature are given in Figure 19, excluding the hold mode at 0.5 kN. Regarding the SS tests at 500 °C and 600 °C (dashed lines in Figure 19) these tests continued beyond 5 mm of crosshead stroke. They were either stopped manually, to avoid equipment failure, or continued until ultimate tensile failure of the straps. In Figure 20, representative axial stress versus axial strain curves is plotted for each SS case, and the results are used to calculate the elastic moduli at the different target temperatures; the curves shown are representative of the responses of the straps under each test condition. Both Figure 19 and Figure 20 portray the tensile behavior of the prestressed straps after 10 min of exposure at the target temperature. The average values and standard deviations for maximum force, UTS, and tensile elastic modulus in the longitudinal (fiber) direction for each target temperature are given in Table 6. 

Calculation of the longitudinal elastic modulus followed the ISO 527-5 standard test method [40], i.e., the strain values used were 0.05% and 0.25% with the corresponding stress values, respectively. In all cases the strain values shown are those obtained using DIC. The data were processed in MATLAB 2019Rb. For the SS tests at 600 °C, the strain values used were 0.05% and 0.5% due to large deformations and deterioration of the DIC white-speckled pattern. Supplementary information on the SS tests is given in Appendix B.

**Figure 21:**
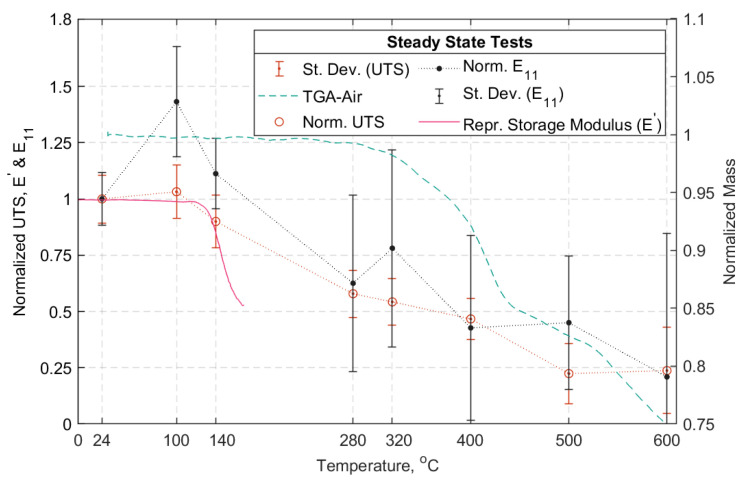
(**Left**): Normalized UTS, normalized elastic modulus (*E_11_*), and normalized storage modulus (representative *E*’) vs. temperature (°C). (**Right**): Normalized mass vs. temperature (°C). SS tests.

Via the SS tests, the influence of temperature on the strength of the straps in the range of 24 to 600 °C was established. This is illustrated in Figure 21, where the average UTS (normalized with respect to the average UTS at ambient temperature) for each temperature is plotted along with its standard deviation. In addition, the impact of temperature on the elastic modulus (*E_11_*) is presented and follows similar trends.

### 4.3. Transient State Thermal (TS) Results

For all three TS tests, the straps were loaded at the target load and remained at that level until their ultimate failure was observed. 

**Figure 22:**
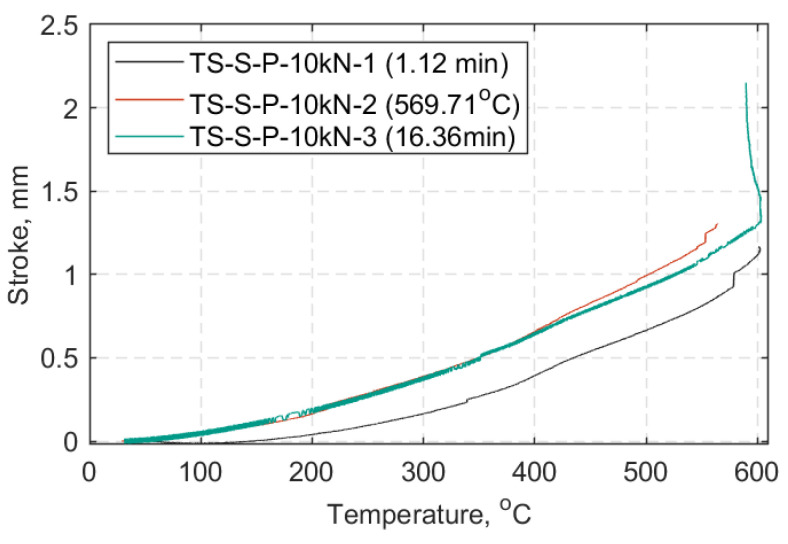
Crosshead stroke in hold mode (mm) vs. temperature (°C): All transient state (TS) cases at 10 kN.

**Figure 23:**
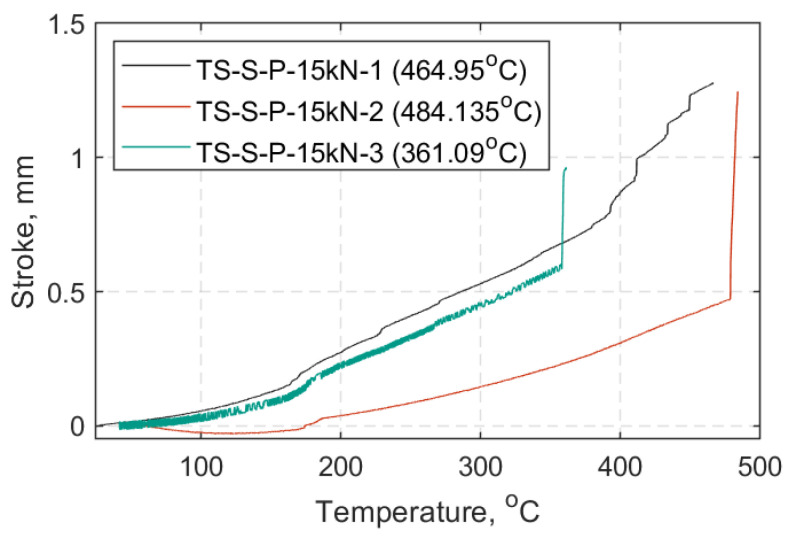
Crosshead stroke in hold mode (mm) vs. temperature (°C): All TS cases at 15 kN.

**Figure 24:**
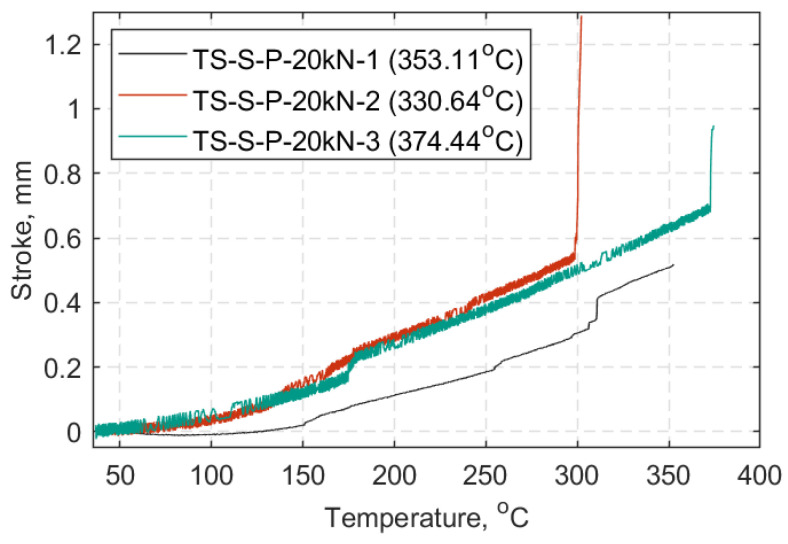
Crosshead stroke in hold mode (mm) vs. temperature (°C): All TS cases at 20 kN.

**Figure 25:**
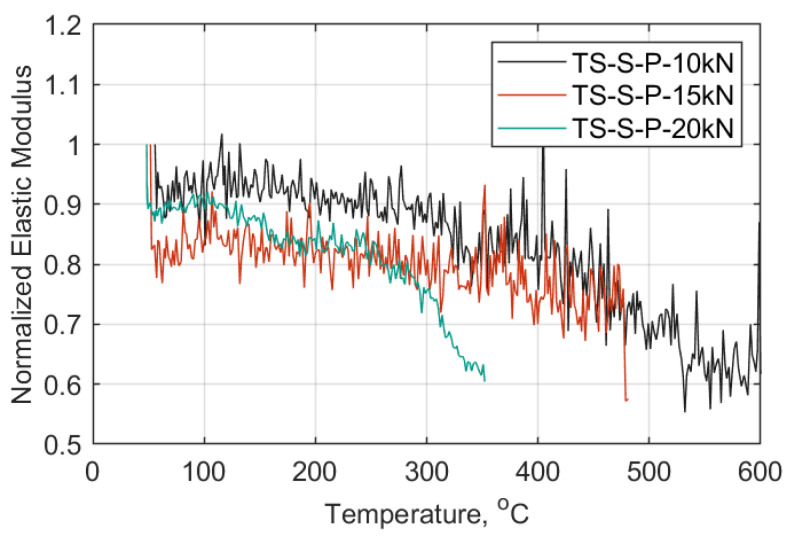
Normalized elastic modulus vs. temperature (°C) representative curves, TS cases.

In Figure 22, Figure 23 and Figure 24 each TS load case is presented in a displacement versus temperature plot (once at the target load). The name notation in the legend for the TS tests is similar to that used in the SS tests, with: TS-Transient State, S-Standard Strap Models, P-Prestressed, 10, 15, or 20 as the target load (kN) in each case, and 1/2/3-test number. When a parenthesis is included, this indicates either the duration (in minutes) of the strap’s ability to sustain the load at 600 °C, or the temperature (°C) at which the strap failed while sustaining the applied load. Representative curves of the elastic modulus progression over temperature for each TS case are also presented in Figure 25. These data represent an approximation of the elastic modulus, since the DIC pattern was significantly deteriorated after reaching 300 °C and noise in the data was inevitable (e.g., out of plane motion, large deformations); further details can be found in Appendix B.

### 4.4. Failure Modes

**Figure 26:**
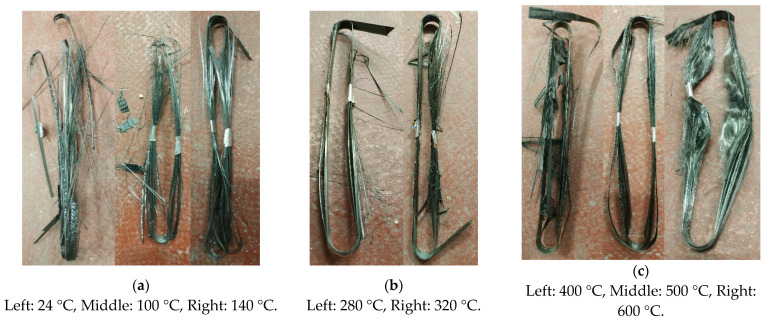
Strap failure modes in SS tests at different temperature levels: (**a**) between 24 and 140 °C, (**b**) at 280 and 320 °C, and (**c**) between 400 and 600 °C.

**Figure 27:**
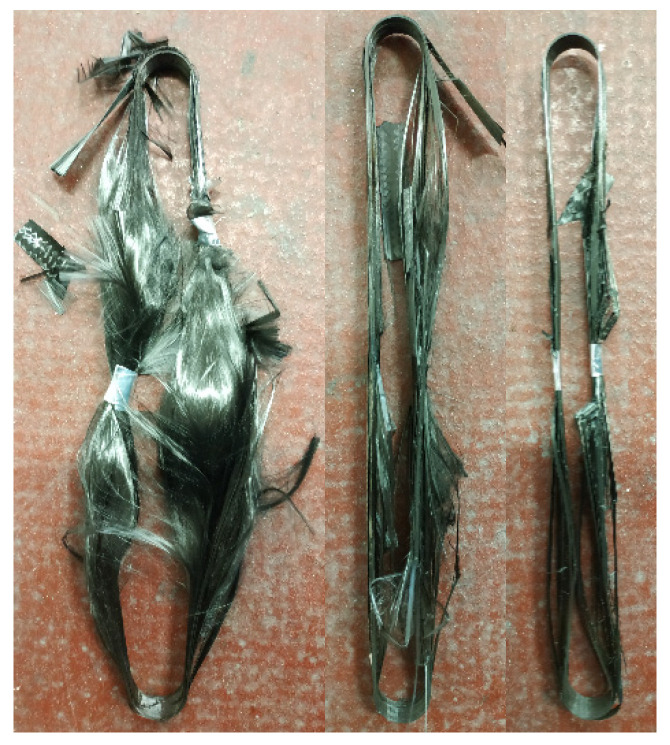
Strap state in TS tests when loaded at 10 kN (**left**), 15 kN (**middle**), and 20 kN (**right**).

For each test case, in-situ pictures of the straps after failure were taken. In this section, representative images for each SS and TS case are presented in Figure 26 and Figure 27, respectively.

**Figure 28:**
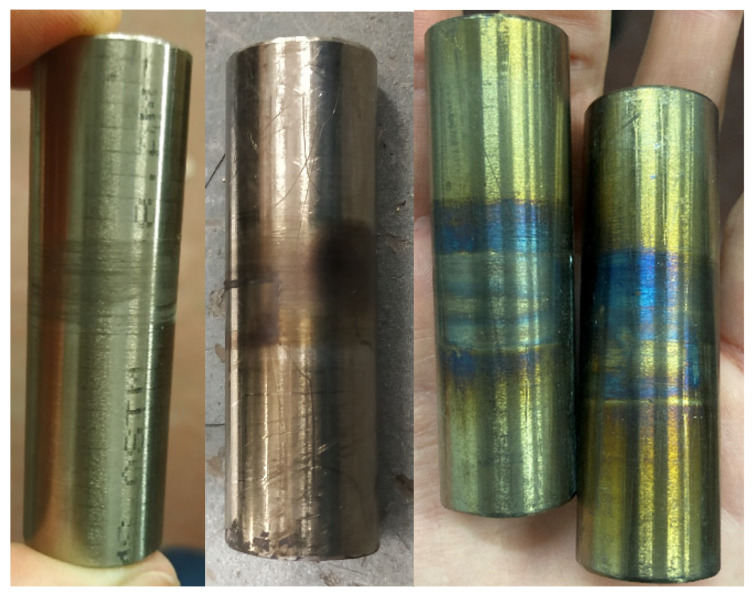
Pin state when exposed up to 140 °C (**left**), up to 500 °C (**middle**), and at 600 °C (**right**).

The state of the titanium pins after exposure at the different temperature levels is shown in Figure 28, and it is evident that the titanium pins were not substantially affected by temperature up to 500 °C, as expected. 

## 5. Discussion

In the SS tests, Thermocouple T2 applied on the shaft surface of the straps nearer to the heating elements and the fan indicated approximately the same temperature as the chamber, when the straps had reached the target temperature. At the same time, Thermocouple 1 (T1) had 1 to 2 °C difference compared to T2; this difference was not evident for temperatures above 320 °C. On the other hand, in the TS tests, T1 and T2 indicated the same temperature (±1 °C) but were always about 5 °C lower than the temperature of the chamber. Regarding Thermocouple 3 (T3) that was bonded on the vertex of the straps, the temperature measurement in both SS and TS tests was consistently up to 100 °C lower than the chamber’s temperature. This temperature mismatch is likely related to the geometry of the grips, the convective conditions within the environmental chamber, and the positions of the thermocouples. 

The main results obtained through the SS and TS tests are given in Section 4.2 and Section 4.3, respectively. Figure 19 summarizes the axial force versus crosshead displacement curves (excluding the load hold mode at 0.5 kN) that best represent each SS temperature case. The maximum force gradually decreased after a peak at 100 °C, as shown in Table 6. A similar trend is evident in the representative stress versus strain curves (Figure 20) where, above 140 °C, the longitudinal modulus gradually decreased as the temperature was increased (see also Table 6). For all the straps tested at 500 and 600 °C, the straps started to progressively unwind after the maximum load was reached, at which point the DIC acquisition was stopped. The force and displacement measurements continued further and were stopped either when the straps could not bear any substantial load or failed, or to avoid equipment damage.

The impact of temperature on the tensile strength of the strap-pin assemblies is presented in Figure 21. It is evident that the straps tested at 100 °C had better performance than those tested at ambient temperature (i.e., 24 °C). The DMTA trace in Figure 7 shows that at 100 °C, the response of the material remains elastic and the viscous part of the response is negligible. This is also evident from the representative storage modulus curve (*E*’) shown in Figure 21. 

At lower temperatures, the stress concentrations were higher than at higher temperatures due to gradual epoxy softening, and since the failure mode tended to be fiber-dominant [27]. This is likely to have allowed for load redistribution between the plies, as the interfacial shear stresses decreased with increasing temperature (up to 100 °C); this could result in an increased overall strength for the CFRP composites. The straps tested at 140 °C (i.e., *T_g_*) had a remaining 0.9 UTS, followed by those tested at 280 and 320 °C that retained more than 55% of the UTS at ambient temperature. This reduction in strength was expected, since the *T_g_* had been exceeded at this point and epoxy softening was likely to have had an impact on stress transfer between individual fibers. At 400 °C, the straps were able to carry about 50% of the UTS at ambient temperature. A sudden loss of strength was evident for temperatures above 500 °C, for which the straps were able to sustain only about 23% of their ambient temperature UTS. This significant loss in strength can most likely be attributed to epoxy decomposition that takes place at temperatures above 410 °C, as shown in Figure 6. Loss of the epoxy resin and progressing exposure of the fibers suggests that the remaining looped fibers carried the loads in part due to the presence of winding friction between them. According to Wang and Kodur [41], who tested CFRP bars (diameter: 9.5 mm, length: 1.35 m, non-continuous fibers) that can be used as internal reinforcement in concrete structures, the critical temperature of the CFRP was defined as the one at which the composite lost 50% of its UTS at ambient temperature. They subsequently suggested that the critical temperature of the CFRP bars was approximately 250 °C based on this (admittedly arbitrary) assumption. With respect to Figure 21, the straps in the current study retained 50% of their ambient temperature UTS at around 365 °C. 

Regarding the longitudinal elastic modulus in the SS tests, at 100 and 140 °C the longitudinal modulus (*E_11_*) appeared to be positively affected when compared against that obtained at 24 °C (see Figure 21). This can most likely be attributed to the potential re-orientation of the slightly wavy fibers in the direction of the load, since the epoxy matrix softens and allows these adjustments between the fibers to take place. Between 280 and 320 °C *E_11_* decreased, and at 400 °C about 40% of the *E_11_* at ambient temperature was retained. At 500 °C, similar retention levels as at 400 °C were exhibited. At 600 °C, approximately 20% of the *E_11_* at ambient temperature was retained; however this is a coarse approximation as only three out of the five tested straps could be analyzed using DIC analysis due to large deformations and excessively deteriorated speckle pattern. For the strain results in the SS tests above 320 °C, out of plane deformations were not incorporated in the strain analysis but *were* evident in the form of exposed fibers combined with unwinding of the inner plies.

The straps’ state after the 10min exposure at the eight different target temperatures is illustrated in Figure 26. In all SS tests, audible crack propagation and/or occasional minor fiber breakages were recorded. The first visible failure mode in all tests was consistently the delamination of the outer ply on the inner side of the wound straps (starting point of the winding). For temperatures up to 320 °C, the final failure of the straps occurred at the vertex area where the straight shaft length met the curvature of the strap around the pin. This was the critical strap region, and is consistent with stress analysis results on tensile loaded straps with pin anchorage [8,20]. At both 24 and 100 °C the straps failed in a sudden and explosive manner, whereas in the range between 140 and 320 °C the straps failed less explosively; likely due to epoxy matrix softening, lower failure loads, etc. In addition, for temperatures between 280 and 320 °C a partial unwinding of the outer plies was observed. Above 400 °C, initial debonding of the outer plies of the strap, followed by progressive debonding and unwinding of the in-between plies was observed. This behavior was attributed to decomposition of the polymer matrix, which was completely decomposed by about 600 °C and led to larger deformations of the straps. Figure 26 (especially above 500 °C) shows that the straps were in a highly deformed state after the tests. 

In the TS tests, the ability of the straps to sustain the 10, 15 and 20 kN load was investigated which is equivalent to approximately 25%, 37%, and 50% of the UTS at ambient temperature, respectively. Figure 22, Figure 23 and Figure 24, show that the straps that were able to reach and remain at 600 °C were those tested at a 10 kN load level (~0.25 UTS). 

As the load level increased from 15 to 20 kN the straps failed earlier, and at lower temperatures. At a 15 kN load level the average failure temperature was about 435 °C (±65 °C) and at a 20 kN load level, it was around 350 ± 21 °C. This is consistent with the main result of the SS tests that gave a temperature of 365 °C for retention of 50% of the ambient temperature UTS (Figure 21). Failure of the straps became more explosive as the load level increased, especially for those straps tested at 20 kN. This behavior was also reported by Zhou et al. [31], who observed that the time to failure of CFRP tendons tested at elevated temperature increased with a decrease of loading level. Comparing the average failure temperatures of the straps (intermediate modulus carbon fibers of type Tenax IMS60) tested at a 0.5UTS level (350 °C) to that of the ultra-high modulus CFRP tendons (diameter: 5.3 mm) tested at 50% of their design tensile strength (409 °C) by Terrasi et al. [30], the straps in the current study failed at lower temperature. This is expected because pitch-based carbon fibers, with higher modulus and higher degree of graphitization, have superior thermal stability than PAN-based normal/intermediate modulus carbon fibers [42]. Despite the lower failure temperature of the model straps at 0.5 UTS level (20 kN) the straps were still able to carry substantial loads for temperatures well above their *T_g_*. 

The temperature progression in the TS tests appeared not to greatly affect the longitudinal elastic modulus up to 330 °C, as shown Figure 25. Ninety percent, 75%, and 65% of the straps’ initial stiffness was retained by the straps at 10, 15, and 20 kN load levels, respectively. Above those temperatures the resistance of the straps due to increasing temperature was more affected. Straps tested at a 20 kN load level were particularly influenced, not only by load level but also by increasing temperature that led to bond loss between the epoxy resin and the fibers, as the number of intermolecular bonds in the resin increased [26,43]. 

The failure modes of the straps in the TS tests were strongly dependent on the load level. Figure 27 shows that for a 20 kN load level the straps failed in an explosive manner, however at lower temperatures of around 350 °C. On the other hand, as the load was lowered to 15 and 10 kN, the straps failed in a less explosive manner and exhibited partial debonding and unwinding of the in-between plies, but failed at higher temperatures. The straps tested at 10 kN were soft and malleable after failure.

Overall, it was observed that the straps’ failure modes and states were in reasonable agreement with observations from the SS tests. It was also observed that, in both SS and TS tests, the shaft of the straps closer to the heating elements was always the more affected by heat. For temperatures above 400 °C, in all cases the gradual removal of epoxy due to decomposition started from the center of the straps and gradually developed towards the pin regions. This was consistent with the reported temperature distribution within the chamber, and was observed for all tested straps. Micromechanical modeling of the strap/pin system exposed to elevated temperatures is considerably more complex than modeling of conventional flat unidirectional coupon tests. Although not discussed in this paper, future work will focus on the development of a time-temperature dependent modeling approach, likely building on Koyanagi’s Simultaneous Fiber-Failure (SFF) model [44].

## 6. Conclusions

The tensile performance of pretensioned, laminated, titanium pin-loaded CFRP straps exposed to elevated temperatures up to 600 °C was investigated and presented. A pretensioning mold able to preload the straps with 10% of their average ultimate failure load at ambient temperature was developed. The carbon fiber reinforced epoxy material of the straps was characterized through thermogravimetric analysis (TGA), dynamic mechanical thermal analysis (DMTA), differential scanning calorimetry (DSC), and standard “burn-off” tests. The glass transition temperature (*T_g_*), the decomposition temperature (*T_d_*), and the fiber volume fraction (*V_f_*) of the CFRP straps were found to be 149.20 ± 1.42 °C, about 410 °C, and 59.33 ± 2.95%, respectively. 

Eight target temperatures, in the range of 24 °C to 600 °C, were chosen (based on results from DMTA and TGA) for the steady state thermal tests. The strength of the straps at different temperature levels was established and it was observed that the load bearing capacity of the pin/strap system gradually decreased with increasing temperature. Fifty percent of the ultimate ambient temperature tensile strength was retained at about 365 °C. The longitudinal tensile elastic modulus (*E_11_*) followed a similar trend, and at 600 °C retained 20% of that at ambient temperature. Ultimately, at temperatures in the range of 500 °C to 600 °C, the straps retained about 25% of their average ambient temperature UTS.

For the transient state thermal tests, the straps were loaded and remained at either 10 kN, 15 kN, or 20 kN that corresponded to approximately 25%, 37%, and 50% of the UTS at ambient temperature, respectively. It was observed that higher loads led to earlier and more explosive failure of the straps. Straps tested at 50% ambient temperature UTS failed at about 350 °C (±21 °C) in the transient thermal tests. The elastic modulus of the straps was affected above 300 °C. Failure modes for both steady and transient state thermal tests have been presented and discussed. 

This study has shown that the model pin-loaded straps performed reasonably well for temperatures up to 360 °C and safely maintained around 50% of their ambient temperature ultimate strength at these temperatures. This has positive practical significance for their potential use as reinforcement components or as reinforcement for concrete or hanger cables in bridges. 

## Figures and Tables

**Table 1:** Materials data [32,33,34].

**Titanium Ti-6Al-4V (Grade 5), Solution Treated Alloy (STA)**	Density (g/cm^3^): 4.43 Tensile Strength—Yield (MPa): 1790 Young’s Modulus (GPa): 114
**IMS60 E13 24K 830tex**	Density (g/cm^3^): 1.79 Tensile Strength (MPa): 5600 Young’s Modulus (GPa): 290
**Epoxy Resin XB 3515/Aradur^®^ 5021**	Density (g/cm^3^): 1.17 Tensile Strength (MPa): 60 ± 1.43Young’s Modulus (GPa): 2.62 ± 0.033

**Table 2:** Volume fractions of matrix and fiber (%) & composite density (g/cm^3^).

Sample	Density (g/cm^3^)	*V_f_* (%)	*V_m_* (%)	*V_v_* (%)
1	1.4559	58.353	35.165	6.482
2	1.4420	56.304	37.105	6.591
3	1.4858	59.922	35.313	4.765
4	1.5520	64.723	33.630	1.648
5	1.4154	57.322	33.276	9.402
Average	1.4702	59.325	34.898	5.778
St. Deviation	±0.0468	±2.952	±1.368	±2.545

**Table 3:** DSC and DMTA results: Glass transition temperature (*T_g_*).

***DSC Samples***			
**Sample**	**Weight, mg**	**Extracted Sample Location**	***T_g_*, Heat Cycle 2 (°C)**
1	19.964	Middle	138.19
	17.348	Curvature	127.19
2	12.870	Middle	140.19
	15.938	Curvature	136.21
3	38.790	Middle	139.22
	38.830	Curvature	133.21
		Average	135.70
		St. Deviation	±4.850
***DMTA Samples***			
**Sample**	**Initial Shape State**		***T_g_*, Peak tanδ Value (°C)**
3PB-1	Sagging-like		149.50
3PB-2	Sagging-like		149.40
3PB-3	Hogging-like		147.20
3PB-4	Hogging-like		150.60
	Average		149.18
	St. Deviation		±1.425

**Table 4:** Pin and strap dimensions.

***Titanium Pin***	
Length (mm)	62
Diameter (mm)	20 ± 0.1
***CFRP Strap***	
Shaft Length (mm) Radius of Curvature (mm)	250 10
Width (mm)	12
Thickness (mm)	1

**Table 5:** Maximum displacement values obtained with DIC and caliper.

DIC Values		Caliper Values	
Sample	δ_max_, mm	Left/Right Gap, mm	Middle Gap, mm
Strap-1	0.744	0.92/0.94	0.95/0.95
Strap-2	0.639	0.86/0.95	0.91/0.91
Strap-3	0.640	0.82/0.86	0.93/0.92
Average	0.674	0.892	0.928
St. Dev.	±0.060	±0.052	±0.018

**Table 6:** Average and standard deviation values of maximum force (kN), ultimate tensile strength (UTS) (MPa), and longitudinal modulus\*E_11_* (GPa), SS tests.

		24 °C	100 °C	140 °C	280 °C	320 °C	400 °C	500 °C	600 °C
*F_max_* (kN)	Average	37.23	39.11	33.11	22.22	20.95	18.19	9.00	9.42
	St. Dev.	±1.92	±4.66	±3.41	±2.12	±2.25	±1.94	±1.13	±1.61
UTS (MPa)	Average	1767.60	1775.78	1548.39	995.94	933.52	803.35	385.40	408.76
	St. Dev.	±159.16	±209.98	±183.00	±104.73	±97.01	±74.01	±51.49	±78.27
*E_11_* (GPa)	Average	142.59	204.20	158.58	89.16	111.40	60.89	64.20	29.84
	St. Dev.	±16.77	±50.26	±24.94	±35.04	±48.77	±25.03	±19.04	±19.01

**Figure A2:**
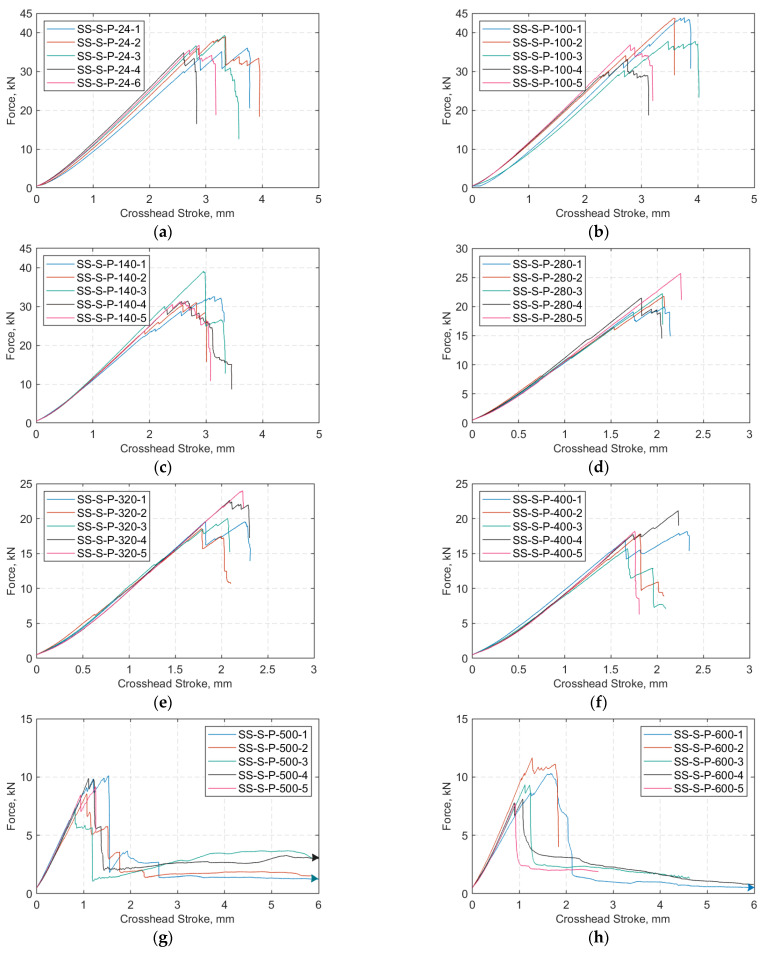
Load (kN) vs. crosshead stroke (mm) for all SS cases: (**a**) 24 °C, (**b**) 100 °C, (**c**) 140 °C, (**d**) 280 °C, (**e**) 320 °C, (**f**) 400 °C, (**g**) 500 °C, and (**h**) 600 °C.

**Figure A3:**
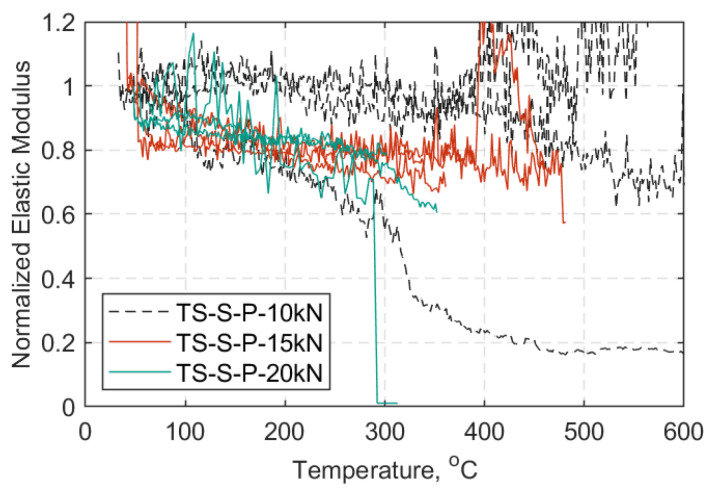
Normalized elastic modulus vs. temperature (°C), TS cases.

## Data Availability

The majority of the data can be found in the PhD thesis to be submitted this year to the University of Edinburgh.

## Appendix A

Additional data to support the material characterization section will be included here. FVF results regarding the weight of the crucibles with and without the composite samples, before and after matrix combustion, are presented in Table A1.

**Table A1:** Crucible and composite weights before and after burn-off (gr).

	*Before Burn-Off*			*After Burn-Off*	
Sample	Empty Crucible (gr)	Composite Only Weight (gr)	Crucible + Composite (gr)	Crucible + Composite (gr)	Composite Only (gr)
1	36.4974	0.7488	37.2462	37.0346	0.5372
2	31.2341	0.8254	32.0595	31.8110	0.5769
3	30.9408	0.8591	31.7999	31.5610	0.6202
4	32.5407	0.6603	33.2010	33.0336	0.4929
5	38.7678	0.8456	39.6134	39.3808	0.6130

±0.0001 scale accuracy; fiber density: 1.79 g/cm^3^; epoxy density: 1.17 g/cm^3^.

The DSC results were all processed in MATLAB R2019a following ASTM D3418-15 standard [38]. In Figure A1, the extrapolated onset and end temperatures are indicated with dashed (red) lines, and the glass transition (midpoint) temperature is the circled value in the zoomed region on the bottom right corner.

## Appendix B

Detailed figures and a table for all the SS and TS cases can be found here. Figure A2 depicts the load vs. displacement curves for all the SS target temperature cases, while in Figure A3 the normalized elastic modulus vs. temperature curves for all the TS target load cases is presented.

The arrows in Figure A2g,h indicate that the tests continued beyond the 6 mm extension and the test was halted either because the straps could not carry any substantial load due to gradual unwinding of the plies or to avoid equipment damage.

**Table A2:** Supplementary results used in SS analysis.

Temp.	Property	1	2	3	4	5	Average	St. Dev.
24 °C	UTS (MPa)	1894.61	1803.63	1934.70	1575.41	1629.63	1767.60	159.16
	*F_max_* (kN)	36.10	39.05	39.33	34.87	36.80	37.23	1.92
	*E_11_* (GPa)	138.34	156.46	115.72	155.91	146.52	142.59	16.77
100 °C	UTS (MPa)	1932.46	2017.30	1706.77	1479.77	1742.57	1775.78	209.98
	*F_max_* (kN)	43.90	43.79	37.83	33.16	36.89	39.11	4.66
	*E_11_* (GPa)	N/A	152.87	203.02	188.25	272.66	204.20	50.26
140 °C	UTS (MPa)	1625.08	1428.75	1837.35	1425.86	1424.88	1548.39	183.00
	*F_max_* (kN)	32.58	31.09	39.12	31.45	31.31	33.11	3.41
	*E_11_* (GPa)	156.08	187.50	163.73	127.02	252.02	158.58	24.94
280 °C	UTS (MPa)	903.16	952.96	1020.58	936.10	1166.90	995.94	104.73
	*F_max_* (kN)	19.94	21.78	22.22	21.47	25.69	22.22	2.12
	*E_11_* (GPa)	92.79	88.25	145.26	56.46	63.05	89.16	35.04
320 °C	UTS (MPa)	895.80	797.21	922.96	1010.05	1041.57	933.52	97.01
	*F_max_* (kN)	19.56	18.58	20.02	22.59	23.98	20.95	2.25
	*E_11_* (GPa)	173.01	60.59	130.99	130.05	62.34	111.40	48.77
400 °C	UTS (MPa)	790.28	828.89	703.78	907.66	786.15	803.35	74.01
	*F_max_* (kN)	18.14	17.80	15.69	21.13	18.18	18.19	1.94
	*E_11_* (GPa)	77.90	80.07	N/A	25.96	59.64	60.89	25.03
500 °C	UTS (MPa)	454.52	368.94	315.66	410.51	377.38	385.40	51.49
	*F_max_* (kN)	10.10	8.55	7.29	9.87	9.19	9.00	1.13
	*E_11_* (GPa)	80.30	64.09	33.18	79.32	64.09	64.20	19.04
600 °C	UTS (MPa)	448.07	516.80	410.88	341.38	326.66	408.76	78.27
	*F_max_* (kN)	10.31	11.65	9.34	8.11	7.73	9.42	1.61
	*E_11_* (GPa)	N/A	N/A	11.68	28.26	49.59	29.84	19.01

Regarding the SS tests, Table A2 provides all the test data used in the SS results summary table (Table 6). It should be noted that the values in red color or the cells denoted with “N/A” were not used in the average and standard deviation calculation due to significant deviation from the overall results or due to missing data, respectively.
